# Spatial patterns of correlation between cortical amyloid and cortical thickness in a tertiary clinical population with memory deficit

**DOI:** 10.1038/s41598-020-77503-2

**Published:** 2020-11-26

**Authors:** Jagan A. Pillai, Mykol Larvie, Jacqueline Chen, Anna Crawford, Jeffery L. Cummings, Stephen E. Jones

**Affiliations:** 1 Lou Ruvo Center for Brain Health, Cleveland Clinic, Cleveland, OH USA; 2 Imaging Institute, Cleveland Clinic, Cleveland, OH USA; 3 Department of Brain Health, School of Integrated Health Sciences, University of Nevada Las Vegas (UNLV), Las Vegas, NV USA; 4 Cleveland Clinic Lou Ruvo Center for Brain Health, Las Vegas, NV USA

**Keywords:** Neuroscience, Diseases of the nervous system, Alzheimer's disease, Dementia, Diagnostic markers, Neurological disorders, Brain

## Abstract

To estimate regional Alzheimer disease (AD) pathology burden clinically, analysis methods that enable tracking brain amyloid or tau positron emission tomography (PET) with magnetic resonance imaging (MRI) measures are needed. We therefore developed a robust MRI analysis method to identify brain regions that correlate linearly with regional amyloid burden in congruent PET images. This method was designed to reduce data variance and improve the sensitivity of the detection of cortical thickness–amyloid correlation by using whole brain modeling, nonlinear image coregistration, and partial volume correction. Using this method, a cross-sectional analysis of 75 tertiary memory clinic AD patients was performed to test our hypothesis that regional amyloid burden and cortical thickness are inversely correlated in medial temporal neocortical regions. Medial temporal cortical thicknesses were not correlated with their regional amyloid burden, whereas cortical thicknesses in the lateral temporal, lateral parietal, and frontal regions were inversely correlated with amyloid burden. This study demonstrates the robustness of our technique combining whole brain modeling, nonlinear image coregistration, and partial volume correction to track the differential correlation between regional amyloid burden and cortical thinning in specific brain regions. This method could be used with amyloid and tau PET to assess corresponding cortical thickness changes.

## Introduction

The 2018 National Institute on Aging and Alzheimer's Association Research Framework emphasizes basing the diagnosis of Alzheimer's disease (AD) on underlying pathologic processes and supporting the clinical diagnosis with the identification of biomarkers grouped into the categories of β amyloid burden, pathologic tau burden, and neurodegeneration; this is known as the AT(N) model^1^. Among the imaging biomarkers, amyloid positron emission tomography (PET) ligand binding (biomarker of Aβ plaques, “A”) is increasingly used clinically, whereas cortical tau PET ligand binding (biomarker of fibrillar tau, “T”) is often used for research. Volumetric structural magnetic resonance imaging (MRI) assessment of neurodegeneration (“N”) is even more widely available and is often the initial biomarker available to a clinician^1^.

The ability of structural MRI to precisely measure regional brain volumes has yielded insights identifying patterns of atrophy related to AD (“N” in the AT[N] model). A “cortical signature” of AD pathology has been reported: more pronounced atrophy in the limbic and heteromodal association cortices, including the hippocampus, posterior and polar temporal lobe, parietal lobe, and portions of the frontal lobe^2,3^. Nonetheless, even as cortical thinning in AD is postulated to be a result of neuronal damage from a combination of neurofibrillary tangles, neuritic plaques, and other pathology^2,4^, the burden of neuritic plaques does not conform to the AD signature areas^5^. Furthermore, cortical thinning is also influenced by other factors including age^6–8^, cognitive reserve^9^, and stage of disease^10–12^.

To better use MRI regional cortical thickness as a biomarker for tracking clinical AD progression while accounting for these above factors, a robust method for coregistration of a PET image (amyloid or tau) to the corresponding MR image is required. An important challenge here is the size scale of the voxels (approximately 1 mm for MRI and 6–8 mm for PET), which creates uncertainty in coregistration. Another challenge involves the nonlinear distortions common with MR images that are more pronounced in the periphery of the imaged volume; these distortions can be several millimeters in size and can be significant compared to normal cerebral cortex, which ranges from 1.0 to 4.5 mm in thickness. Imprecise coregistration can lead to cortex in one modality being compared to white matter in the other modality. It is therefore not surprising that although multiple studies have tracked MRI cortical changes to cross-sectional and longitudinal PET amyloid burden and assessed the relationship between these factors and cognitive decline^13–28^, two key questions related to precise quantification of regional cortical information remain: (1) Is the relationship between PET amyloid burden and cortical thickness linear or nonlinear^13,17^ and (2) does this relationship vary across individual cortical regions? To answer these questions, precise measurement of spatially coincident cortical thickness and amyloid is required, for which we have developed improved image processing tools; nevertheless, methodological challenges remain an obstacle to accurate correlation of amyloid and tau PET signal with regional cortical thickness.

To this end, we designed a novel image processing method to reduce data variance and improve the sensitivity of detection of the cortical thickness–amyloid correlation in individual cortical regions. The key elements of the method, detailed in the next section, involve whole brain modeling, nonlinear image coregistration, modified partial volume correction, and 3 T imaging. While these technical advances have been applied separately in previous work by others, this study is the first to demonstrate the utility of combining these methods. We next used our novel method to investigate the spatial patterns of correlations between regional amyloid burden and regionally coincident cortical thinning. This research is unique in applying this method fully to all brain regions, in particular the cortex, and also in including cerebrospinal fluid (CSF) regions as controls (as there should be no relationship between CSF standardized uptake value ratios [SUVR] and CSF volumes).

Previous research in prodromal AD found a pronounced correlation between tau PET ([18F]flortaucipir) and cortical thickness in temporal and parietal regions, whereas the correlation between amyloid PET ([^18^F]flutemetamol) and cortical thickness was less region specific^4^. We therefore used our novel image processing method to assess concomitant amyloid PET and MRI images from a cross-sectional cohort of memory clinic patients to test the hypothesis that the degree of correlation between amyloid burden and cortical thickness is highest in the temporoparietal neocortical regions where early AD pathology is noted (also described by tau PET). We also hypothesized that evaluation of the relationship between regional amyloid burden and cortical thickness would allow us to estimate the accuracy of using structural MRI metrics to infer the burden of amyloid and tau as measured using amyloid and tau PET.

## Methods

### Overview of approach

Achieving accurate correlation of cortical atrophy and amyloid burden throughout the brain depends on obtaining a near-exact coregistration of PET to MRI, such that the cortical ribbon from each modality shares considerable overlap (≥ 50%). Thus, if the mean cortical thickness is 2–3 mm, the accuracy of coregistration needs to be on order of 1 mm. To achieve this accuracy, three sources of potential error must be addressed. The first is the considerable difference in spatial resolution between the modalities (approximately 1 mm for MRI and 6–8 mm for PET). The second involves the markedly different tissue contrast characteristics of the two modalities; although many algorithms can accurately coregister two scans using the same modalities, the accuracy is understandably lower when coregistering scans from different modalities, such as PET and MRI. The third source of error involves subtle but widespread spatial distortions of MRI. While not apparent to the naked eye, these distortions can alter measured distances in the brain by more than 1 mm when compared with measurements obtained with less-distorted modalities such as CT. In this study, we addressed the first two errors by constructing a PET model derived from the brain region segmentation of the MRI data, which should achieve similar spatial resolution and permit coregistration between like modalities (PET to PET). To address the third error, we used a higher order coregistration (both affine and nonlinear) between the modeled PET and PET. Additionally, the final values of cortical amyloid burden were adjusted for partial volume correction (PVC), a goodness-of-fit parameter was calculated, and final cortical amyloid values for all cortical parcels were compared to cortical thickness in a patient cohort.

### Patients

This retrospective study was approved by the Cleveland Clinic Institutional Review Board, and all protected health information requirements were strictly followed and all methods were carried out in accordance with relevant guidelines and regulations. Informed consents were also waived by the Cleveland Clinic Institutional Review Board, in this retrospective study with minimal risk. Using a patient registry from the Cleveland Clinic Lou Ruvo Center for Brain Health, a tertiary memory clinic, we identified 75 patients who underwent both a brain florbetapir PET scan and a head MRI scan (following the ADNI protocol available at adni.loni.usc.edu) between January 2013 and January 2017; both scans were performed as part of a diagnostic evaluation of neurocognitive deficits. Of these 75 patients, 55 had a consensus diagnosis of AD dementia, 5 had mild cognitive impairment (MCI) likely from AD (all amyloid positive), and 15 were amyloid negative (as determined from the radiologist’s report on the amyloid PET scan) and had diagnoses of subjective memory complaints that were likely non-neurodegenerative and were included in the analysis to establish an amyloid-negative baseline. The clinical consensus diagnoses were made by a team of behavioral neurologists, neuropsychologists, and neuroradiologists and were based on clinical criteria for AD dementia^29^ and for MCI due to AD^30^. *APOE ε* 4 genetic information was not available for all patients and was not included in the analysis.

### MRI acquisition

Volumetric T1-weighted images were obtained at 3 T using Siemens MRI scanners (Skyra, Prisma, or Trio, depending on the site; Siemens Medical Systems, Erlangen, Germany). The volumetric sequences were obtained using the ADNI MPRAGE protocol with the following acquisition parameters: repetition time/echo time/inversion time = 2300/2.98/900; flip angle = 9°; bandwidth = 240 Hz/pixel; 240 × 256 matrix; 160 slices; voxel size = 1 × 1 × 1.2 mm; scan time = 9:14; and 32-channel head coil. Images were acquired in the sagittal plane with 1.2-mm slice spacing and in-plane voxel size of 1.0 × 1.0 mm.

### PET acquisition

All PET images were obtained using a combined PET/computed tomography (CT) system (Siemens Biograph mCT 20 or 40, depending on the site) and were analyzed retrospectively. The voxel sizes were 0.78 × 0.78 × 2.0 (thick) mm for CT images and 2.12 × 2.12 × 2.03 (thick) mm for PET images. The Biograph 20 scanner used a 16-cm axial field of view, with reconstruction performed using a 3D OSEM algorithm (6 iterations, 21 subsets, 2-mm Gaussian filter, without resolution modeling, and with time-of-flight information). The Biograph 40 scanner used a 21-cm axial field of view, with reconstruction performed using a 3DOSEM algorithm (6 iterations, 24 subsets, 3-mm Gaussian filter, with resolution modeling, and without time-of-flight information). The amyloid PET radiotracer used was ^18^F florbetapir, administered intravenously at a prescribed dose of 10.0 mCi (delivered doses ranged from 8.1 to 12.0 mCi). A 15-min image acquisition was obtained approximately 50 min after injection. Standard data corrections were applied (randoms, deadtime, normalization, decay, scatter, and CT-derived attenuation correction).

### MRI segmentation

For all patients, FreeSurfer 6.0 processing^31,32^ was performed on the volumetric T1-weighted sequence, which provided segmentation of each hemisphere’s 34 cortical parcels, 34 subcortical white matter parcels, and 97 remaining parcels of deep gray matter structures, other white matter structures, brainstem, cerebellum, and ventricles. A small number of voxels remained as unsegmented structures with common labels. Because this confounded later modeling, these small areas were relabeled to match those of the most common nearest neighbor segmentations, using custom software written in IDL (Interactive Data Language, Harris Geospatial Solution, Boulder, CO, USA). A total of 165 volumes for a model were fit to the PET images. A binary mask was formed around the parcellated brain using a 3-voxel dilation. All subsequent coregistrations between PET and MRI data were conducted with the MRI space used as the reference.

### Preprocessing and initial coregistration of PET, CT, and MRI

The head frame was first removed from CT images because it confounded coregistration algorithms. Because we used a combined PET/CT scanner, the native PET and CT images were oriented in a common physical space; however, because of the large difference in voxel sizes, the native PET image was upsampled to match the image space of the CT scan. A rigid coregistration was performed between the MRI T1-weighted image and CT images using FLIRT from FSL^33,34^, with 6 degrees of freedom and a normmi search cost. The resulting transformation matrix was applied to the native PET image, thereby producing a new image (hereafter referred to as the “PET image”) that was coarsely coregistered to the MR image. Because of the intrinsic higher spatial resolution of the MR image, the MR image was used as a reference image in all subsequent analyses, and all other transformations were made on the PET image.

### Construction of a PET model from segmented MRI

A PET model was constructed using the entire parcellation from FreeSurfer output of MR images. This enabled us to make comparisons of the same modality (PET images with modeled PET), thus allowing for fine-tuned coregistration using higher order techniques. The model was constructed by first assigning the mean PET activity in the corresponding location of the PET image for each cortical parcel. However, a simple correspondence is not possible because of partial volume averaging caused by the relatively larger point spread function of PET, wherein the activity originating from one parcel will “bleed in” to adjacent parcels (an effect that will be corrected at a later stage). The typical full width at half maximum (FWHM) of this spread can range from 6 to 8 mm, much larger than the typical cortical thickness of 2–3 mm. Thus, this blurring was modeled independently for all segmented parcels using a Gaussian convolution with a FWHM of either 6 or 8 mm, assuming each parcel had a uniform distribution of amyloid signal. These values were chosen to reflect the range of observed resolution from PET images, which combines the intrinsic resolution of the hardware and some degree of superimposed additional blurring due to any patient motion. A modeled PET image could then be constructed if the value of uniform amyloid signal was provided for each parcel. PET and MRI could then be compared using the surrogate of a PET model, and a more refined coregistration could be performed by removing the nonlinear distortions from the MRI scan.

### Fine-tuned coregistration

To fine-tune the coregistration between the PET image and the PET model, two methods were used: an affine (degrees of freedom = 12) using FLIRT, and a nonlinear coregistration using Advanced Normalization Tools^35–37^. The FLIRT routine used a cost function of leastsq, with a constrained search in all three dimensions of − 20 to 20. The Advanced Normalization Tools allowed us to bring the gyration and sulcation patterns into more precise agreement.

### Partial volume correction

Because the point spread function of PET is larger than the typical thickness of cortex, amyloid activity measured in one region necessarily includes activity partially spreading or averaging from adjacent regions. If the spread function is known, it should be possible to account for this spread and compute PET maps without any spread. A number of PVC techniques have been described for this use^38^; these methods increase the accuracy of cortical amyloid measurements but at the cost of increased noise. For this study, we adopted the geometric transfer matrix method, which was first described by Rousset et al.^39^ and Labbe et al.^40^. In short, given a set of N parcels covering the entire brain and a spread function characterized by a parameter such as the FWHM, a square matrix can be constructed (in our case, 165 × 165) for which the partial spread or loss from any parcel to all other parcels is computed. The matrix equation can then be solved to obtain the intrinsic (i.e., nonspread) values at each parcel. Routine least-squares methods can be used to invert the matrix, but we found that these results were often unstable, resulting in parcels with nonphysical values (e.g., negative or unrealistically high). In effect, a superior fit could be obtained in some brain parcels at the expense of a negative SUVR in other parcels. A solution was to use a constrained least-squares routine that could place lower and upper limits on the results, for which we used the lsqlin routine in MATLAB (MathWorks, Natick, MA, USA), using a lower limit constraint of 0 SUVR in any parcel (i.e., cannot be negative). The final result was a set of 165 numbers representing the mean amyloid activity in all parcels. Because there is no practical calibration for the absolute amyloid PET radiotracer uptake in the brain, all values were normalized to a common region to generate SUVRs, and for this research, the brainstem was used. Alternative calculations (not presented) tested other regions such as the cerebellum, which is commonly used, but goodness-of-fit parameters were marginally better using the brainstem. All cortical parcels have a corresponding subcortical white matter parcels; therefore, the mean ratio of cortical activity to subcortical white matter activity was computed. All calculations were performed using IDL.

### Measuring goodness of fit

To quantify the accuracy of coregistration methods, a normalized root-mean-square difference between two images was used. In particular, for an image comparing N voxels, the following equation was used:

1 $$fit = \frac{{\sqrt {mean(PET - PET_{model} )^{2} } }}{{mean\left( {PET + PET_{model} } \right)/2}},$$

where the mean is computed over all voxels. The fit was computed for all models over the entire brain or only the cortex. A value close to 0 would indicate a good fit; a value close to 1 would indicate a poor fit. Goodness-of-fit results are presented in Supplementary Information.

### Application to patients and statistical analysis

Three different models were applied to images from all patients: (1) rigid coregistration with no PVC; (2) affine coregistration with PVC; and (3) nonlinear coregistration with PVC. Each model yielded best-fit values for normalized amyloid activity in the 165 parcels used in this study. Of these, 68 cortical parcels could be compared with the mean cortical thickness values provided by FreeSurfer. Computing these values for each patient then allowed us to further analyze the relationship between cortical amyloid and thickness. Each parcel could be evaluated for significance of correlation (described below), and the overall performance of each model could be judged by the number of parcels with a significant correlation. A drawback to this measure is its assumption that there is an underlying relationship between SUVR and cortical thickness; there may be some parcels for which no such relationship actually exists. Thus, although this may not be the best technique for evaluating any of the three described methods individually, it is likely a reasonable way to compare the relative merits of the three methods against each other.

Analysis of the ventricles formed a useful control for testing the PVC method, as the ventricular size would not be expected to have any correlation with soluble amyloid contained within the CSF (i.e., there should not be a significant correlation between ventricular SUVR and ventricular volume).

Statistical analyses were performed using the IMSL_MULTIREGRESS routine in IDL to create multiple linear regression models and the IMSL_PARTIAL_COV routine to calculate partial correlation coefficients. The first statistical analysis performed a simple linear regression to calculate the Pearson correlation coefficient between the cortical thickness and amyloid uptake, yielding a correlation coefficient ρ and its associated *p* value. A second statistical analysis used a multivariable linear regression including the clinical variables of education, age, sex, and Montreal Cognitive Assessment (MoCA) score with the model assuming

2 $${\text{Cortical amyloid }}\sim \, \left( {\text{cortical thickness}} \right) \, + \, \left( {{\text{education}}} \right) \, + \, \left( {{\text{age}}} \right) \, + \, \left( {{\text{sex}}} \right) \, + \, \left( {{\text{MoCA}}} \right)$$

yielding an overall correlation coefficient and regression coefficients with associated *p* values for the 5 dependent variables. Following multiple comparisons across 68 FreeSurfer cortical regions (using both hemispheres), the false discovery rate (FDR) was controlled using the Benjamini–Hochberg procedure^41^, using an FDR of 0.1 to yield *p* < 0.018. An independent sample t-test was used to compare continuous variables, and Fisher’s exact test was used for categorical variables. A difference with a two-tailed probability of *p* < 0.05 was considered statistically significant (Null hypothesis was that the degree of correlation between amyloid burden and cortical thickness is the same in all regions).

A pipeline diagram of the analysis method is provided in the supplementary material as Supplementary Fig. 2.

## Results

### Patients

A summary of demographic features of the 75 patients included in the final data analysis is provided in Table 1. These patients were subcategorized into 3 groups roughly reflecting the severity of dementia, as demarked by MoCA scores: 0–10 (*n* = 11); 11–19 (*n* = 31); and 20–30 (*n* = 33).

**Table 1:** Patient demographics based on 4 clinical diagnoses.

Characteristic	AD Dementia (*n* = 55)	MCI-AD (*n* = 5)	Amyloid negative subjective memory complaints (*n* = 15)	Total (*n* = 75)
Age, years	74.8 (6.2)	70.6 (12.4)	74.6 (4.5)	74.5 (6.4)
Education, years	14.7 (3.6)	15.6 (2.6)	16.8 (3.3)	15.2 (3.5)
MoCA score	16.3 (6.6)	21.2 (4.5)	22.0 (3.8)	17.8 (6.5)
Sex, female	32 (58%)	3 (60%)	4 (27%)	39 (52%)

Unless otherwise noted, numbers in parentheses represent the standard deviation from the mean.

*AD* Alzheimer disease, *MCI* mild cognitive impairment, *MoCA* Montreal Cognitive Assessment.

### Image coregistration

**Figure 1:**
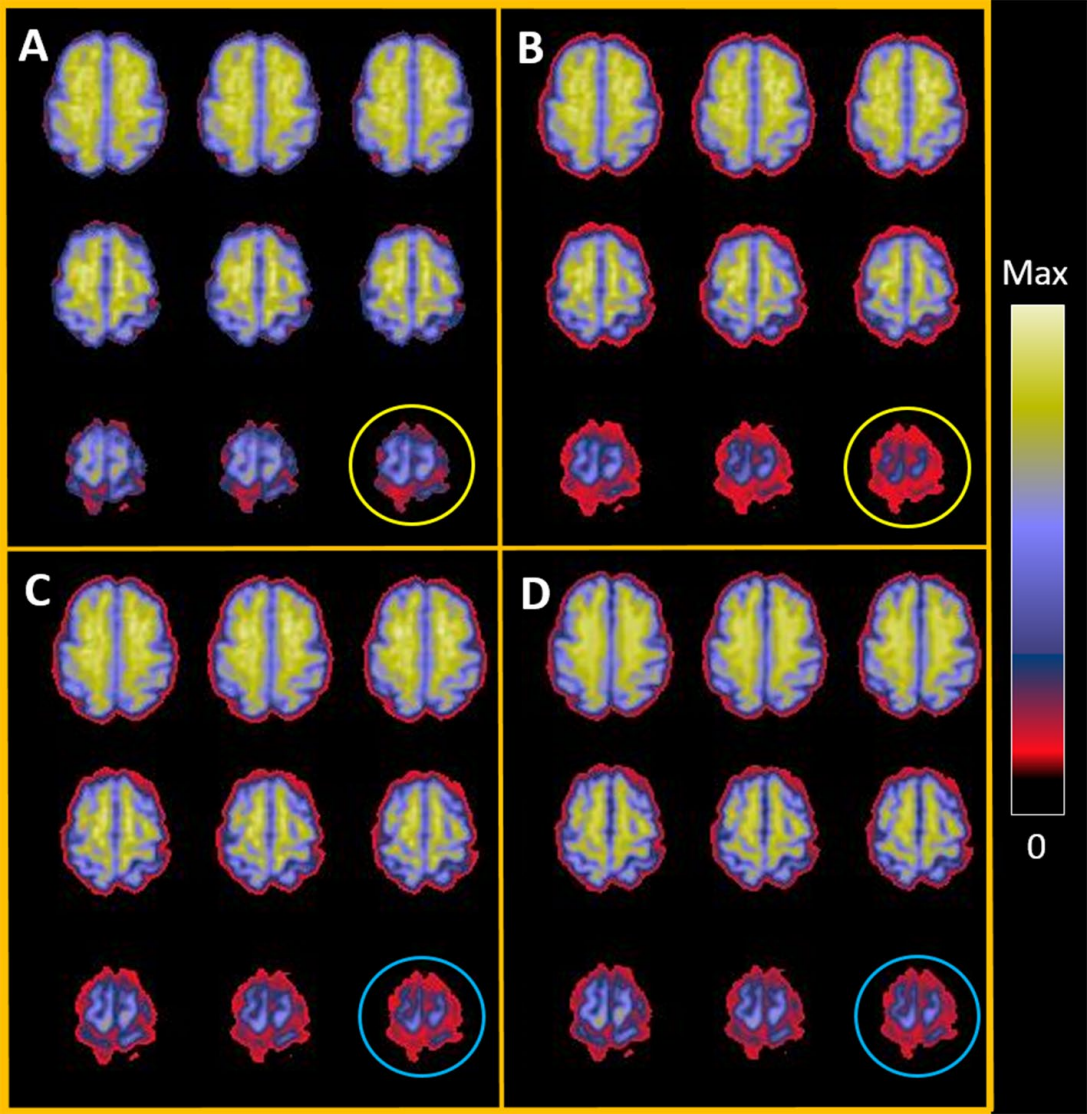
Sample images of PET data, coregistration, and PET models. Images show (**A**) PET data after rigid coregistration of native PET data to MRI space; (**B**) initial MRI-based model of PET data derived from FreeSurfer parcels, using values from (**A**) as inputs, with no PVC; (**C**) nonlinear coregistration of native PET data to MRI space; and (**D**) final MRI-based PET model using input values from (**C**) and incorporating PVC. The correspondence between (**A**) and (**B**) was grossly adequate but insufficient for details close to the cortical ribbon. Differences are best appreciated along the margins of features (e.g., near the apex, as shown in the orange circles). The correspondence between (**C**) and (**D**) was greatly improved with the use of nonlinear coregistration and PVC (see blue circles). The colored display reflects the SUVR, with the normalized magnitude indicated by the color bar, scaled between the maximum value (which was close to SUVR = 3.0) and zero. *MRI* magnetic resonance imaging, *PET* positron emission tomography, *PVC* partial volume correction, *SUVR* standardized uptake value ratio.

Figure 1 shows imaging data from a single patient and demonstrates the utility of higher order coregistration and PVC. The best correspondence was seen between nonlinear coregistered PET and the PET model using PVC, particularly in the details of cortical overlap. Additional details comparing our novel MR image processing to previous methods are provided in Supplementary Information, which includes results about goodness-of-fit measurements. For example the goodness of fit parameter (Eq. 1) across the entire brain assuming a FWHM of 8 mm was 0.163 for nonlinear coregistered PET with PVC, whereas 0.388 for a rigid coregistration with no PVC.

### Association between cortical amyloid and cortical thickness

**Figure 2:**
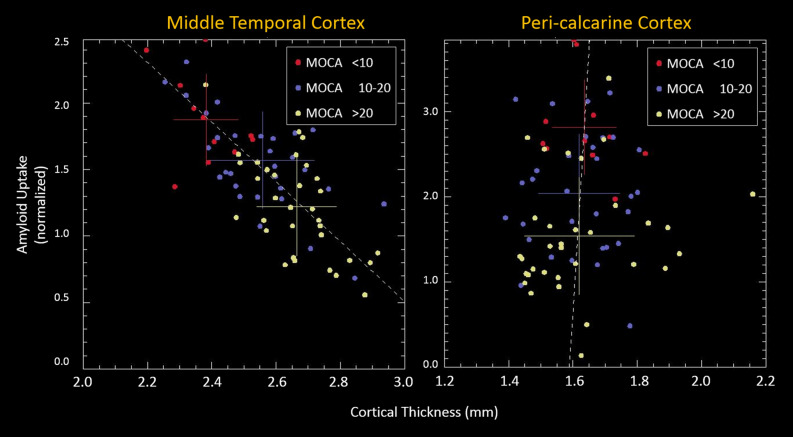
Scatter plot demonstrating cortical thickness and cortical amyloid uptake as measured by SUVR. Left: a region of high Pearson correlation (R = 0.69; *p* = 7 × 10^–12^) in the middle temporal gyrus. Right: a region of low Pearson correlation (R = 0.07; *p* = 0.53) in the pericalcarine cortex. The patients are color-coded into three groups reflecting the degree of degenerative disease (stratified by MoCA score), with crosshairs for each group representing means and standard deviations. Red data are from patients with MoCA score ≤ 10; blue data are from patients with MoCA score of 11–19; and yellow data are from patients with MoCA score ≥ 20. *MoCA* Montreal Cognitive Assessment, *SUVR* standardized uptake value ratio.

Figure 2 shows scatter plots of regional amyloid uptake versus cortical thickness for two cortical parcels: the middle temporal cortex and the pericalcarine cortex. In each case, the group with the lowest MoCA score demonstrated the highest amyloid uptake. In the middle temporal lobe, this was associated with significant cortical thinning; in the pericalcarine cortex, there was no change in cortical thickness. These two plots are exemplary of the ends of the relationship spectrum, i.e., one plot shows a strong relationship between cortical thickness and amyloid uptake, whereas the other shows no relationship. In particular these examples show a range of Pearson coefficient between a high correlation (R = 0.69; p = 7 × 10–12) and no correlation (R = 0.07; p = 0.53).

For each brain parcel, a simple linear regression model and multivariable linear regression were used to measure the association between cortical amyloid and cortical thickness (Table 2). In the simple regression model, the temporal lobe had the greatest number of regions with highly significant correlations (three regions), followed by the parietal lobe (also with three regions, but less significant compared with the temporal lobe). In the multivariable model, the variable with the strongest correlation was the MoCA score (with 28 parcels out of 34 being significant), followed by education (20 parcels) and cortical thickness (11 parcels). All brain regions except the entorhinal, parahippocampal, and posterior cingulate regions showed significant correlation of MoCA with amyloid SUVR. Analysis of the lateral ventricles showed no significant correlation between ventricular SUVR and ventricular volume when using a nonlinear coregistration with a constrained PVC (r = 0.04, p = 0.66). A significant correlation resulted when using either a rigid coregistration without PVC (r = − 0.72, p < 0.001) or nonlinear coregistration with an unconstrained PVC (r = − 0.38, p < 0.001) (see Supplementary Information and Supplementary Fig. 2).

**Table 2:** Statistical significance of correlations between cortical amyloid and cortical thickness for all FreeSurfer cortical parcels, using a simple model (regression of regional amyloid vs cortical thickness) and a multivariable model (comparing regional amyloid as a function of cortical thickness, age, education, sex, and MoCA score).

Parcel	Simple model	Multivariable model				
	Cortical thickness	Cortical thickness	Age	Education	Sex	MoCA
Frontal pole	− 0.352 [− 0.813, 0.109]	− 0.331 [− 0.773, 0.112]	0.002 [− 0.012, 0.016]	− 0.016 [− 0.043, 0.011]	− 0.111 [− 0.294, 0.073]	− 0.019** [− 0.005, − 0.034]
Lateral orbitofrontal	− 0.473 [− 1.107, 0.160]	− 0.109 [− 0.668, 0.500]	0.008 [− 0.004, 0.021]	− 0.021 [− 0.045, 0.003]	− 0.095 [− 0.259, 0.068]	− 0.027**** [− 0.039, − 0.014]
Medial orbitofrontal	− 0.349 [− 1.232, 0.534]	− 0.123 [− 0.910, 0.664]	0.008 [− 0.009, 0.025]	− 0.040* [− 0.073, − 0.008]	0.017 [− 0.210, 0.244]	− 0.035**** [− 0.053, − 0.018]
Pars orbitalis	− 0.512 [− 1.139, 0.114]	− 0.468 [− 0.973, 0.037]	0.004 [− 0.010, 0.018]	− 0.024 [− 0.050, 0.002]	− 0.250** [− 0.430, − 0.070]	− 0.033***** [− 0.047, − 0.019]
Pars triangularis	− 0.850 [− 2.011, 0.310]	− 0.750 [− 1.69, 0.178]	0.010 [− 0.009, 0.029]	− 0.036 [− 0.073, 0.001]	− 0.174 [− 0.427, 0.079]	− 0.053***** [− 0.073, − 0.033]
Pars opercularis	− 1.295* [− 2.422, − 0.148]	− 0.531 [− 1.518, 0.455]	0.007 [− 0.012, 0.026]	− 0.043* [− 0.079, − 0.007]	0.029 [− 0.218, 0.277]	− 0.050***** [− 0.070, − 0.030]
Rostral middle frontal	− 0.552 [− 1.765, 0.661]	− 0.673 [− 1.606, 0.260]	0.005 [− 0.013, 0.022]	− 0.043* [− 0.077, − 0.009]	0.027 [− 0.205, 0.259]	− 0.056***** [− 0.074, − 0.038]
Caudal middle frontal	− 1.177** [− 2.013, − 0.342]	− 0.922** [− 1.563, − 0.281]	0.006 [− 0.010, 0.023]	− 0.042** [− 0.073, − 0.010]	0.101 [− 0.113, 0.314]	− 0.053***** [− 0.070, − 0.036]
Superior frontal	− 0.953* [− 1.743, − 0.162]	− 0.837* [− 1.486, − 0.189]	0.002 [− 0.014, 0.018]	− 0.041** [− 0.071, − 0.010]	0.081 [− 0.129, 0.292]	− 0.043***** [− 0.059, − 0.026]
Paracentral	− 0.281 [− 0.939, 0.376]	0.027 [− 0.514, 0.568]	0.007 [− 0.009, 0.022]	− 0.032* [− 0.061, − 0.003]	− 0.012 [− 0.212, 0.187]	− 0.044***** [− 0.060, − 0.029]
Precentral	0.061 [− 0.574, 0.696]	0.233 [− 0.269, 0.734]	0.007 [− 0.006, 0.021]	− 0.037** [− 0.063, − 0.012]	− 0.082 [− 0.257, 0.094]	− 0.036***** [− 0.050, − 0.023]
Postcentral	0.238 [− 0.678, 1.153]	0.276 [− 0.415, 0.967]	0.002 [− 0.012, 0.016]	− 0.036* [− 0.064, − 0.008]	− 0.206* [− 0.394, − 0.018]	− 0.043***** [− 0.058, − 0.028]
Superior parietal	− 0.704 [− 1.447, 0.038]	− 0.128 [− 0.779, 0.523]	0.006 [− 0.009, 0.021]	− 0.032* [− 0.061, − 0.002]	− 0.011 [− 0.210, 0.188]	− 0.041***** [− 0.057, − 0.024]
Inferior parietal	− 2.527***** [− 3.343, − 1.711]	− 1.853***** [− 2.617, − 1.090]	0.015 [− 0.001, 0.031]	− 0.039* [− 0.069, − 0.009]	− 0.047 [− 0.255, 0.160]	− 0.034*** [− 0.052, − 0.016]
Supramarginal	− 1.811*** [− 2.735, − 0.887]	− 1.020* [− 1.804, − 0.236]	0.011 [− 0.006, 0.027]	− 0.042** [− 0.073, − 0.010]	− 0.098 [− 0.315, 0.119]	− 0.044***** [− 0.062, − 0.027]
Precuneus	− 1.403** [− 2.281, − 0.525]	− 0.764 [− 1.627, 0.099]	0.003 [− 0.017, 0.023]	− 0.035 [− 0.073, 0.003]	0.069 [− 0.194, 0.332]	− 0.040*** [− 0.062, − 0.018]
Cuneus	− 0.359 [− 1.389, 0.671]	− 0.197 [− 1.098, 0.704]	0.022* [0.003, 0.042]	− 0.023 [− 0.061, 0.015]	− 0.090 [− 0.344, 0.165]	− 0.043**** [− 0.063, − 0.023]
Peri-calcarine	0.410 [− 0.863, 1.684]	0.466 [− 0.593, 1.525]	0.022 [− 0.001, 0.046]	− 0.002 [− 0.048, 0.043]	0.009 [− 0.302, 0.319]	− 0.068***** [− 0.092, − 0.044]
Lateral occipital	− 1.213*** [− 1.915, − 0.512]	− 0.781* [− 1.454, − 0.108]	0.011 [− 0.004, 0.026]	− 0.035* [− 0.064, − 0.005]	− 0.032 [− 0.229, 0.164]	− 0.033**** [− 0.050, − 0.017]
Fusiform	− 0.725**** [− 1.063, − 0.387]	− 0.285 [− 0.726, 0.156]	0.001 [− 0.010, 0.011]	− 0.019 [− 0.040, 0.002]	− 0.069 [− 0.213, 0.076]	− 0.019* [− 0.034, − 0.005]
Lingual	− 0.317 [− 0.964, 0.329]	− 0.003 [− 0.616, 0.610]	0.007 [− 0.006, 0.019]	− 0.012 [− 0.037, 0.013]	− 0.121 [− 0.287, 0.044]	− 0.026*** [− 0.040, − 0.013]
Temporal pole	− 0.138 [− 0.377, 0.100]	− 0.057 [− 0.304, 0.190]	0.000 [− 0.012, 0.0120]	− 0.015 [− 0.037, 0.008]	− 0.104 [− 0.258, 0.051]	− 0.008 [− 0.020, 0.005]
Superior temporal	− 1.863***** [− 2.446, − 1.281]	− 1.113*** [− 1.732, − 0.494]	0.010 [− 0.004, 0.023]	− 0.026* [− 0.051, − 0.001]	− 0.004 [− 0.177, 0.168]	− 0.028*** [− 0.043, − 0.013]
Middle temporal	− 1.777***** [− 2.205, − 1.350]	− 1.366***** [− 1.879, − 0.853]	0.014** [0.004, 0.025]	− 0.018 [− 0.038, 0.003]	− 0.044 [− 0.182, 0.094]	− 0.010 [− 0.024, 0.003]
Inferior temporal	− 0.939**** [− 1.369, − 0.509]	− 0.502* [− 0.944, − 0.059]	0.009 [− 0.001, 0.020]	− 0.025* [− 0.046, − 0.004]	− 0.011 [− 0.154, 0.131]	− 0.022** [− 0.035, − 0.009]
Entorhinal	− 0.058 [− 0.206, 0.091]	− 0.016 [− 0.183, 0.150]	0.003 [− 0.008, 0.014]	− 0.015 [− 0.034, 0.005]	0.025 [− 0.110, 0.161]	− 0.002 [− 0.014, 0.009]
Parahippocampal	− 0.162 [− 0.407, 0.084]	− 0.087 [− 0.342, 0.168]	− 0.002 − 0.013, 0.009]	− 0.020* [− 0.041, − 0.000]	− 0.016 [− 0.154, 0.121]	− 0.010 [− 0.021, 0.001]
Insula	− 0.487 [− 0.989, 0.014]	− 0.207 [− 0.716, 0.303]	0.004 [− 0.008, 0.015]	− 0.029* [− 0.052, − 0.006]	0.099 [− 0.059, 0.257]	− 0.020** [− 0.034, − 0.006]
Transverse temporal	− 0.716 [− 1.455, 0.023]	− 0.426 [− 1.094, 0.243]	0.023* [0.001, 0.045]	− 0.046* [− 0.087, − 0.006]	0.081 [− 0.195, 0.356]	− 0.044**** [− 0.066, − 0.023]
Banks	− 3.085***** [− 4.125, − 2.045]	− 2.003*** [− 3.144, − 0.863]	0.017 [− 0.006, 0.040]	− 0.048* [− 0.093, − 0.004]	0.147 [− 0.159, 0.454]	− 0.045** [− 0.073, − 0.017]
Caudal anterior cingulate	− 0.316 [− 0.821, 0.188]	− 0.206 [− 0.670, 0.258]	0.009 [− 0.009, 0.026]	− 0.038* [− 0.072, − 0.005]	0.124 [− 0.108, 0.355]	− 0.029** [− 0.047, − 0.011]
Rostral anterior cingulate	− 0.414 [− 0.959, 0.130]	− 0.491* [− 0.959, − 0.023]	0.015 [− 0.005, 0.035]	− 0.046* [− 0.085, − 0.007]	0.069 [− 0.198, 0.336]	− 0.044**** [− 0.065, − 0.023]
Isthmus cingulate	− 0.661** [− 1.133, − 0.189]	− 0.552* [− 1.050, − 0.054]	− 0.004 [− 0.022, 0.013]	− 0.022 [− 0.055, 0.011]	0.012 [− 0.217, 0.241]	− 0.013 [− 0.032, 0.006]
Posterior cingulate	− 0.153 [− 0.721, 0.415]	− 0.184 [− 0.742, 0.374]	0.002 [− 0.019, 0.024]	− 0.040 [− 0.082, 0.002]	0.085 [− 0.201, 0.371]	− 0.022 [− 0.044, 0.001]

Values in brackets are 96% confidence intervals. *p* values: *, < 0.05–0.01; **, < 0.01–0.001; ***, < 0.001–0.0001; ****, < 0.0001–0.00001; *****, < 0.00001; all other *p* values are > 0.05.

*MoCA* Montreal Cognitive Assessment.

Bold denotes *p* value < 0.05.

**Figure 3:**
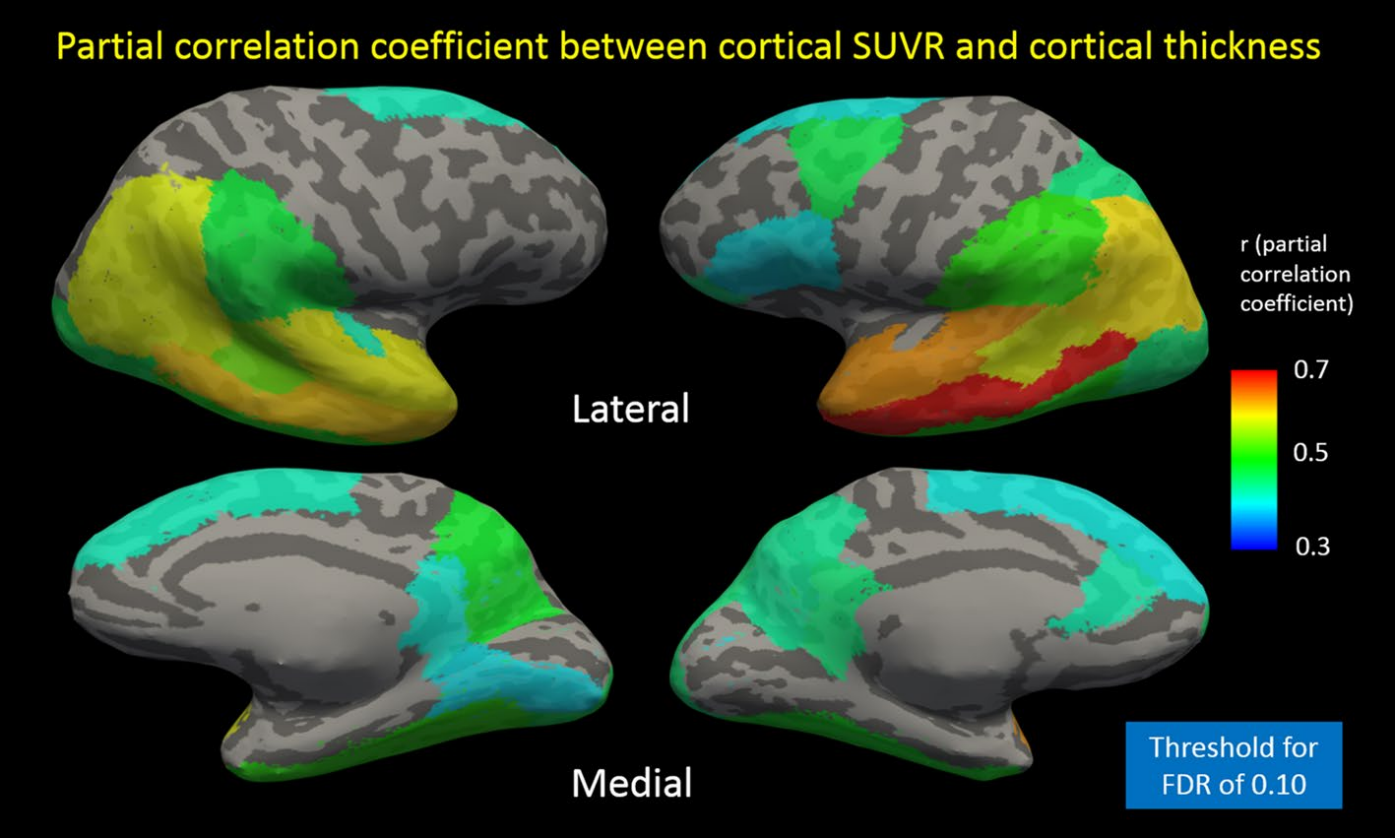
Cortical surface overlay showing the partial correlation coefficient between cortical amyloid and cortical thickness. Partial correlation coefficient is shown for each cortical parcel after adjustments were made for age, education, sex, and MoCA score. Displayed are the medial and lateral views of each hemisphere. Using a FDR of 0.1 yielded a *p* value threshold of 0.037, corresponding to a correlation coefficient threshold of 0.23. *FDR* false discovery rate, *MoCA* Montreal Cognitive Assessment.

**Figure 4:**
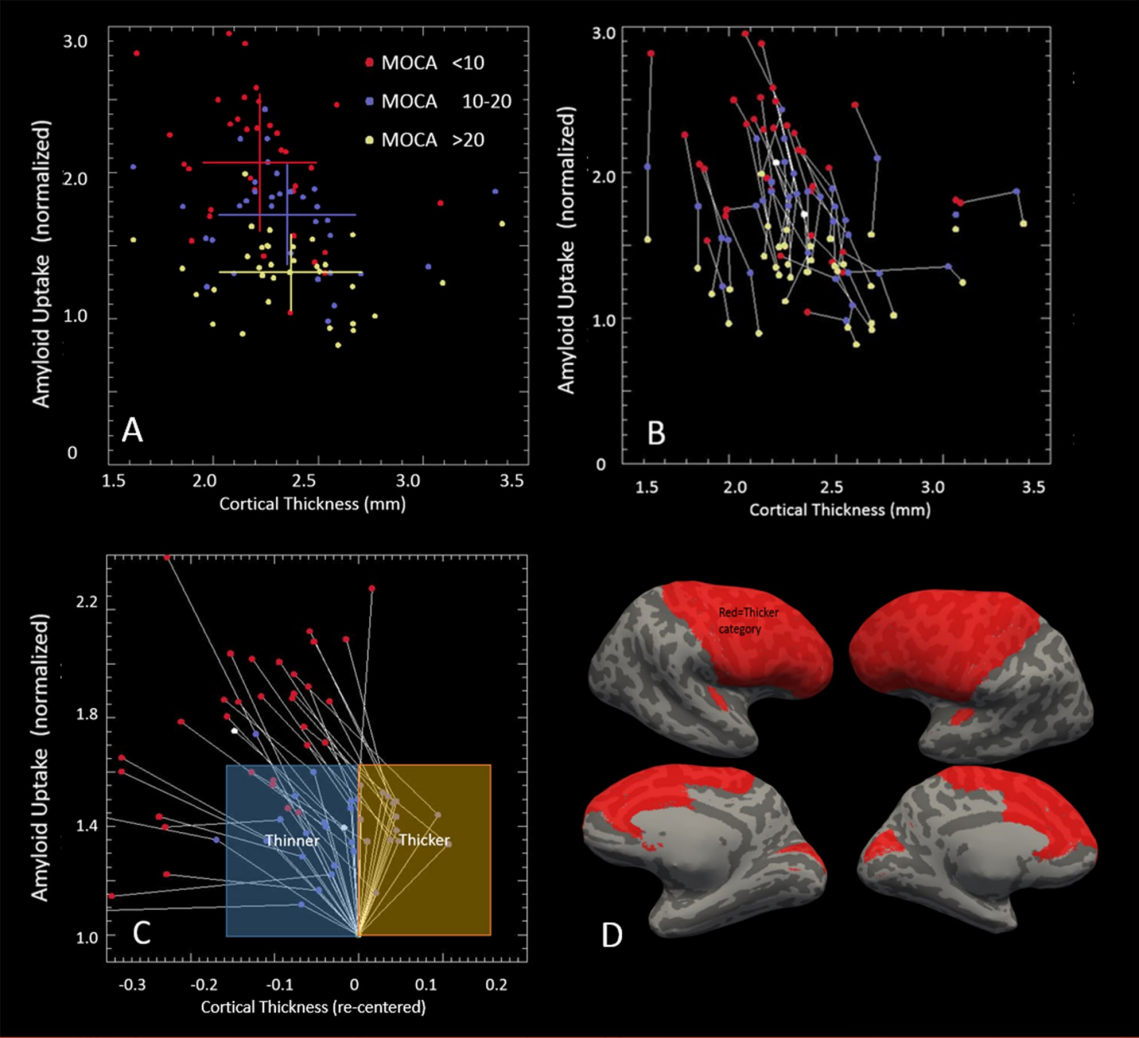
Patterns of amyloid–cortical thickness values extended to all cortical parcels. (**A**) Scatter plot for all 34 cortical parcels (each an average of both hemispheres). Each parcel was subanalyzed separately classified by the MoCA score of the patient (red: MoCA score 0–10; blue: MoCA score 11–19; yellow: MoCA score 20–30). Each dot is an average of the cortical thickness and amyloid (SUVR) for each parcel, for all patients in the group indicated by the color. Overlaid crosshairs show the means and standard deviations for the three groups. (**B**) Scatter plot of the same data points from (**A**) but with overlaid lines connecting the points belonging to the same parcel. (**C**) Similar scatter plot as shown in (**A**) and (**B**) but with each parcel normalized to a common cortical amyloid SUVR (1.0) and cortical thickness value (0.0) for the high MoCA score group (yellow circles). The parcels were grouped into 2 categories (thicker or thinner) depending on how the high MoCA group (yellow circles) compared with the middle MoCA group (blue circles). This shows the trend of decreased cortical thickness from high MoCA to low MoCA groups. (**D**) Surface image of an inflated brain using data from (**C**), showing the spatial variation from a simple dichotomization from (**C**) (i.e., between high MoCA to medium MoCA. The regions of less thinning generally occur in the frontal parenchyma, Heschl’s gyrus, and calcarine sulcus. *MoCA* Montreal Cognitive Assessment, *SUVR* standardized uptake value ratio.

Figure 3 shows an inflated surface image of both hemispheres, with a colored overlay indicating the partial regression coefficient. The threshold of significance for each region was 0.23, derived from an FDR of 0.1 and yielding a *p*-value of 0.037. The regions of significance corresponding to AD signature regions included the medial and lateral parietal lobes, temporal lobes, and prefrontal lobes (left more than right). Of note, regions outside of the AD signature regions, including the fusiform gyri and lateral occipital regions, were also significant. Averaged over all parcels, the earliest stages of the disease (subjective memory complaints, MCI) were associated with increased amyloid SUVR but stable cortical thickness, whereas the later stages of the disease (AD dementia) were associated with correlated change (increased amyloid in parallel with cortical thinning) (Fig. 4). The average cortical SUVR and cortical thickness values for each of the three patient groups in Fig. 4A are shown in Table 3. AD signature regions of the parietal and temporal lobes were associated with the most change in cortical thickness across AD stages, while the frontal lobes were associated with a smaller degree of change (Fig. 4D).

**Table 3:** Cortical thickness and amyloid SUVR for patient groups. Values are means ± standard deviations.

	Patient groups classified by MoCA score		
	0–10 (*n* = 11)	11–19 (*n* = 31)	20–30 (*n* = 33)
Cortical thickness, mm	2.22 ± 0.27	2.35 ± 0.32	2.37 ± 0.34
Amyloid SUVR	2.07 ± 0.47	1.71 ± 0.34	1.32 ± 0.26

*MoCA* Montreal Cognitive Assessment, *SUVR* standardized uptake value ratio.

The number of regions with significant correlations also varied depending on the coregistration and PVC methods used, with more accurate coregistration yielding more brain regions demonstrating statistical significance (Table 4). In general, the left hemisphere showed more significant correlations than the right (17 versus 13, using a nonlinear coregistration with constrained least-square fit PVC), and there was an increasing number of significant relationships with increasing goodness of fit (30 for nonlinear coregistration with constrained least-square fit PVC, compared to 17 for rigid coregistration with no PVC).

**Table 4:** Number of significant cortical parcels seen with various coregistration and PVC methods using a 0.10 FDR, derived from a partial correlation analysis between cortical amyloid and cortical thickness (after adjustments were made for age, education, and sex).

Coregistration	PVC used	Left hemisphere	Right hemisphere	Hochberg *p* value for 0.10 FDR
Rigid	No	9	8	0.012
Affine	Yes	17	11	0.040
Nonlinear	Yes	17	13	0.037

Note the nonlinear method was chosen for overall final analysis of the data.

*FDR* false discovery rate, *PVC* partial volume correction.

## Discussion

In this cross-sectional study, we used a novel MRI-PET image processing method to investigate in better detail the cortical thickness as measured by MRI with the corresponding amyloid burden as measured by amyloid PET. We found that increasing accuracy of coregistration methods yields more brain regions of significant correlation between the MRI and PET measures of interest. Linear relationships were notable in the fusiform gyri and lateral occipital regions in our study as reported in prior neuropathology studies^5^, rather than in the medial temporal or the AD cortical signature regions. Our results suggest that the sigmoidal nature of cortical thinning reported across AD stages previously^42,43^ in fact varies depending on regional brain amyloid burden. Additionally, age, sex, and education altered the linear relationship between amyloid and cortical thickness, suggesting that these variables (in addition to stage of disease) also relate to the correlation between cortical thickness and amyloid burden in AD signature regions, thus limiting cortical thickness utility to track disease progression.

Hippocampal volume derived from structural MRI is a robust and commonly used metric of neurodegeneration (“N”) in the AT(N) model. Although hippocampal volume is often used as a clinical biomarker of AD in clinical trials, our results highlight the severe limitations of inferring corresponding amyloid burden (“A”) using hippocampal volume or cortical thickness in AD signature regions as a proxy for inferring amyloid severity burden (such as with amyloid PET or CSF amyloid assays) across the AD spectrum. Our results demonstrated linear correlation between cortical thickness and amyloid burden in the fusiform gyrus and lateral occipital cortices but not the medial temporal regions regardless of disease severity.

Most previous studies of AD used a global measure of amyloid burden, and early studies reported variations in the regions of significant correlation^4,13–16^. In an earlier study that used local measures of amyloid and atrophy in cognitively normal elderly participants, a correlation was identified in the parietal regions, including the precuneus, but not in the medial temporal regions^17^. Local measures of amyloid and cortical atrophy in our study of a memory clinic cohort, using a novel image coregistration technique, again found that cortical thickness in the AD signature regions did not demonstrate the strongest correlation with amyloid.

There are several possible reasons for these results. First, our study population displayed a wide range of AD severity, ranging from subjective cognitive impairment to AD dementia. Second, there were differences between our study and previous research in MRI processing and analytical techniques. Finally, atrophy in the AD signature regions may be sensitive to multiple factors beyond amyloid burden alone. Indeed, recent research demonstrating the appearance of neurodegeneration in the absence of Aβ and tau has challenged the Aβ- and tau-centric model of AD pathophysiology^44–46^.

Metrics derived from structural MRI are widely used in memory clinics worldwide; thus, the strength of our study is that it was performed in a clinical cohort of patients with memory changes. Accordingly, the robust cortical atrophy patterns and correlation with amyloid burden are likely to be replicated in typical clinical populations. Additionally, rather than using a global measure of amyloid burden, we used a novel image analysis method to improve upon limitations in methods previously used for these analyses, including improved coregistration of MRI and PET images.

The study design was cross-sectional; no longitudinal data were obtained. Given that there was temporal progression of all patients from high MoCA (20–30) to middle (11–19) to low (0–10) scores, with each group having differences in mean cortical thickness and mean SUVRs, longitudinal trends may be inferred. A cross-sectional cohort of patients with a wide range of MoCA scores may be helpful in exploring longitudinal behavior in a scatter plot of SUVR versus cortical thickness. In the context of this study, the data suggest that a decrease in cognitive scores (high to mid-range MoCA score) is correlated with increased cortical SUVR but little change in cortical thickness; the change from mid-range to low MoCA score is revealed by both increased cortical SUVR and decreased cortical thickness. In this cross-sectional evaluation of different stages of the disease, amyloid SUVR changes are noted without significant cortical thinning. True longitudinal data would be required to verify this interpretation.

The limitations of this study reflect the challenges involved in characterizing cortical thickness, which can be affected by multiple variables. Variabilities in the manifestation of amyloid burden and atrophy in different disease subtypes limit the current results to the common amnestic form of AD, which was typical of the amyloid-positive patients in this cohort. Because we used local correlations in our analysis, these results, like those from previous studies, are not sensitive to network effects, whereby amyloid accumulation in a given region contributes to cortical atrophy in a distinct non-coincident region. Tau PET was not available for our study cohort; although neurofibrillary tangles may contribute to atrophy in some brain regions, this factor could not be assessed in this study. However, the image analysis methods used in this study can be used to improve the analysis of tau burden and cortical thickness in studies using tau PET.

In addition to neurodegeneration related specifically to Alzheimer pathology, the aging brain is subject to other acquired injuries, most notably vascular disease, and these injuries likely contribute at least additively and possibly synergistically to the neurodegeneration of AD^47^. Patients with vascular dementia were excluded from this study, and so this factor could not be characterized. Study patients may have differed in other possibly confounding factors affecting atrophy, including the presence and severity of metabolic conditions such as diabetes^48^ and nutritional status. Finally, the presence of hippocampal sclerosis^49^ and mixed pathology such as combinations of AD, Lewy body pathology, and vascular pathology^50^ are also known to affect rates of cognitive decline, but these conditions could not be reliably identified in our cohort without neuropathology confirmation. It would be helpful to further evaluate these findings in a larger autopsy-confirmed cohort of individuals to overcome the likelihood of a false positive (type 1) error.

In conclusion, with the use of novel imaging analysis methods to enable better coregistration for regional cortical analysis, we identified a robust regional cortical atrophy signature that linearly tracks regional amyloid SUVRs in patients with AD. We found that fusiform gyrus and lateral occipital cortical thickness, not medial temporal cortical thickness, had the strongest linear relationship with regional amyloid SUVRs. Our novel image processing methods could also be used for tau PET to better assess the correlation between regional tau burden and cortical thickness. These analyses could contribute to improved understanding of the imaging correlates of AD pathology.

## Supplementary information

Supplementary Figure 1.

Supplementary Figure 2.

Supplementary Information.

## Data Availability

The datasets analyzed during the current study are available from the corresponding author upon reasonable request pending IRB and institutional approval.

## Abbreviations

AD: Alzheimer’s disease
CSF: Cerebrospinal fluid
CT: Computed tomography
FDR: False discovery rate
FWHM: Full-width at half-maximum
MCI: Mild cognitive impairment
MoCA: Montreal Cognitive Assessment
MRI: Magnetic resonance imaging
PET: Positron emission tomography
PVC: Partial volume correction
SUVR: Standardized uptake value ratio
