# Supplementary figures and images for: Spatial patterns of correlation between cortical amyloid and cortical thickness in a tertiary clinical population with memory deficit
Source: Sci Rep. 2020 Nov 26;10:20717. doi: 10.1038/s41598-020-77503-2 (PMC7693188; doi:10.1038/s41598-020-77503-2)

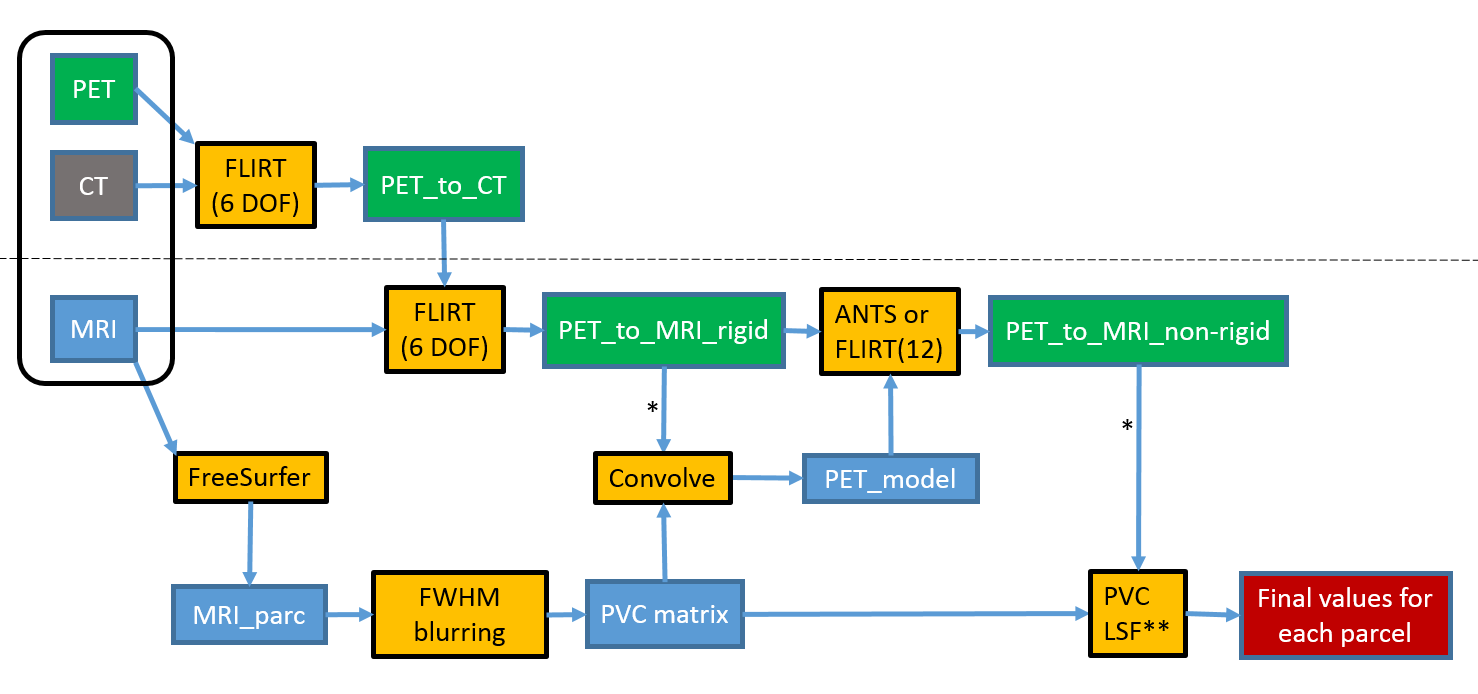

Supplement: Supplementary file 1 — Supplementary Figure 1. [file 41598_2020_77503_MOESM1_ESM.tif]

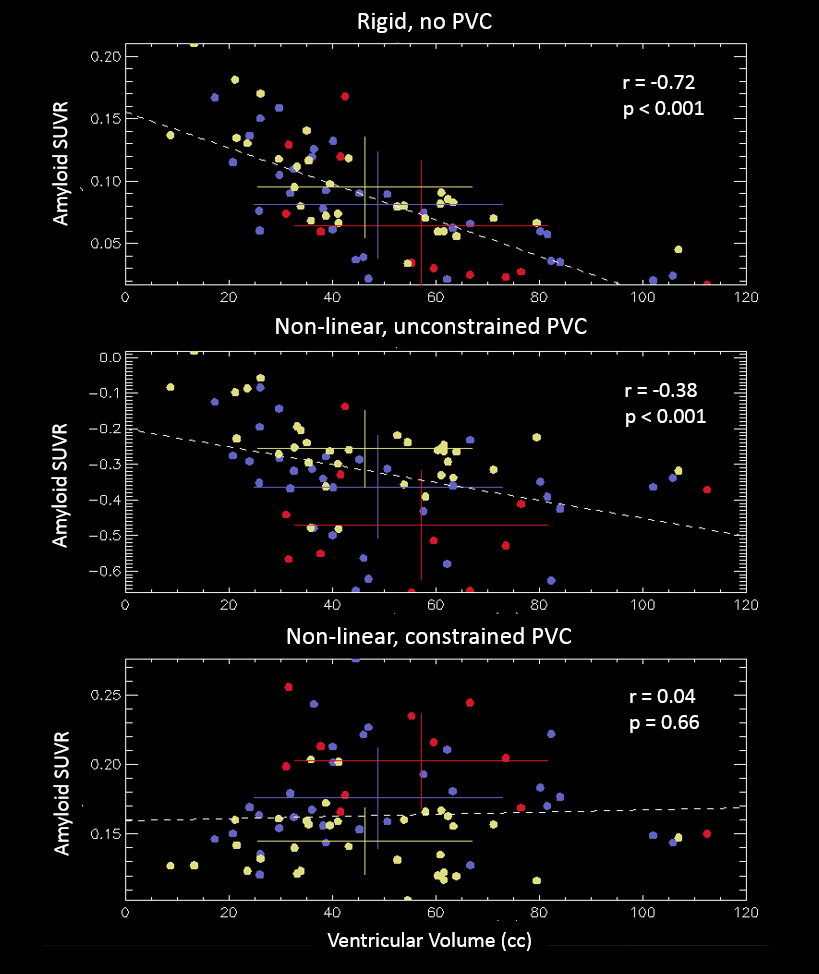

Supplement: Supplementary file 2 — Supplementary Figure 2. [file 41598_2020_77503_MOESM2_ESM.tif]
