# Supplementary material for: Spatial patterns of correlation between cortical amyloid and cortical thickness in a tertiary clinical population with memory deficit
Source: Sci Rep. 2020 Nov 26;10:20717. doi: 10.1038/s41598-020-77503-2 (PMC7693188; doi:10.1038/s41598-020-77503-2)
Supplement: Supplementary file 3 — Supplementary Information. [file 41598_2020_77503_MOESM3_ESM.docx]

**Spatial patterns of correlation between cortical amyloid and cortical thickness in a tertiary clinical population with memory deficit**

Jagan A. Pillai, Mykol Larvie, Jacqueline Chen, Anna Crawford, Jeffery L. Cummings, Stephen E. Jones

**Supplementary Information**

**Pipeline of data analysis**

**Supplementary Figure 1** shows a block diagram of the analysis pipeline used in this paper. The three native input images of the brain are FDG PET, CT (concurrently obtained with the PET), and volumetric MRI (typically obtained at a different timepoint), as shown in the top left and bounded by a box. All PET images (both native and postprocessed) are shown in green boxes. Similarly, all MRI data are shown in blue boxes. Computational operations on images and other data are shown in orange boxes. All of the image data below the dashed line remain in the native MRI space in terms of voxel size and field of view, implying that all PET-derived images below the dashed line are now converted to this same computational space. The asterisks denote image data that have been reduced from all the voxels in the brain volume to reflect the smaller number of FreeSurfer parcels, of which 165 total are used in this analysis. That is, each parcel in the brain is related to the average value of all the individual voxels within its region. In short, there is a succession of coregistrations, starting with a crude rigid registration to approximate an upsampled PET image to the MRI, followed by a fine nonrigid coregistration (either nonlinear or affine) of the PET data to the MRI-derived PET model. Included is a PVC, which uses either a constrained or unconstrained least-square fit.

**Goodness of fit of coregistration between PET and MRI model**

The acquired native PET image is intrinsically blurred because of the resolution limits of the PET methodology, and there is some degree of superimposed additional blurring due to any patient motion. To more accurately compare the PET images to a PET model, the model needs to include blurring, or smoothing. Because the degree of blurring is not fixed and can vary between scanners and settings, two different smoothing factors were used that could be expected to address the range of image blurring. Three different goodness-of-fit calculations were performed: (1) smoothing of the PET model to FWHM = 6 mm results for all patients; (2) smoothing of the PET model to FWHM = 8 mm results for all patients; and (3) using both 6-mm and 8-mm smoothing for each patient but selecting the one smoothing factor with the best fit. The purpose of this last calculation was to account for variability in the best fit for individual patients, which is due at least in part to motion blurring. In general, marked improvement of the fit was seen with increasing sophistication of the method, with the best fit seen for nonlinear coregistration using a constrained least-square fit PVC method (**Supplementary Table S1**). The fit was better for the cortex than for the entire brain. The fit was mildly improved with FWHM = 8 mm, with little further improvement seen when adopting the calculation for individual variations.

**Association between cortical amyloid and subjacent white matter amyloid**

Using the FreeSurfer cortical and adjacent subcortical white matter parcels, we computed the ratio of cortical amyloid to subjacent white matter amyloid for all parcels. We found that the amount of cortical amyloid was less than the amount of amyloid in the subjacent white matter across all patients, with a ratio ranging from 0.83 to 0.87 depending on the model (**Supplementary Table S2**). More accurate models provided a higher ratio, indicating that raw amyloid PET images overestimate the reduction in normal cortex. In patients with lower MoCA scores, who tend to have more advanced disease, the ratio increased to 0.9 for the rigid coregistration model but reached near unity (1.05) for more advanced models, suggesting that with careful alignment and PVC, amyloid deposition becomes uniform across the cortical gray matter and white matter.

**Correlation of amyloid signal in ventricles compared with ventricular size**

In addition to all FreeSurfer cortical parcels, the measures such as those shown in Figure 2 were determined for all 165 parcels used in the model, including white matter regions, basal ganglia, and all CSF–containing regions. The value of including the lateral ventricles is that they form a highly sensitive criterion for accuracy of the PVC models, since florbetapir SUVR in CSF should not be expected to vary with ventricle volume. This criterion was routinely violated when PVC was not used, likely because the region of a small ventricle is proportionately more susceptible to spread from adjacent white matter than the region of a large ventricle. A similar violation showed the need for a constrained PVC in which the mathematical routine would not permit outliers such as regions with negative activation or unreasonably large activity; this constraint was required for ventricular CSF amyloid to be independent of ventricular volume.

**Supplementary Figure 2** shows scatter plots similar to those in **Figure 2**, except for the lateral ventricles. There are three plots resulting from three variants of models: rigid coregistration with no PVC, nonlinear coregistration with unconstrained PVC, and nonlinear coregistration with constrained PVC (which is the final model used in the final analysis of results in this paper). The first two plots show a statistically significant correlation between ventricular SUVR and ventricle volume, which is nonphysical. Only the last model shows a correlation that is zero.


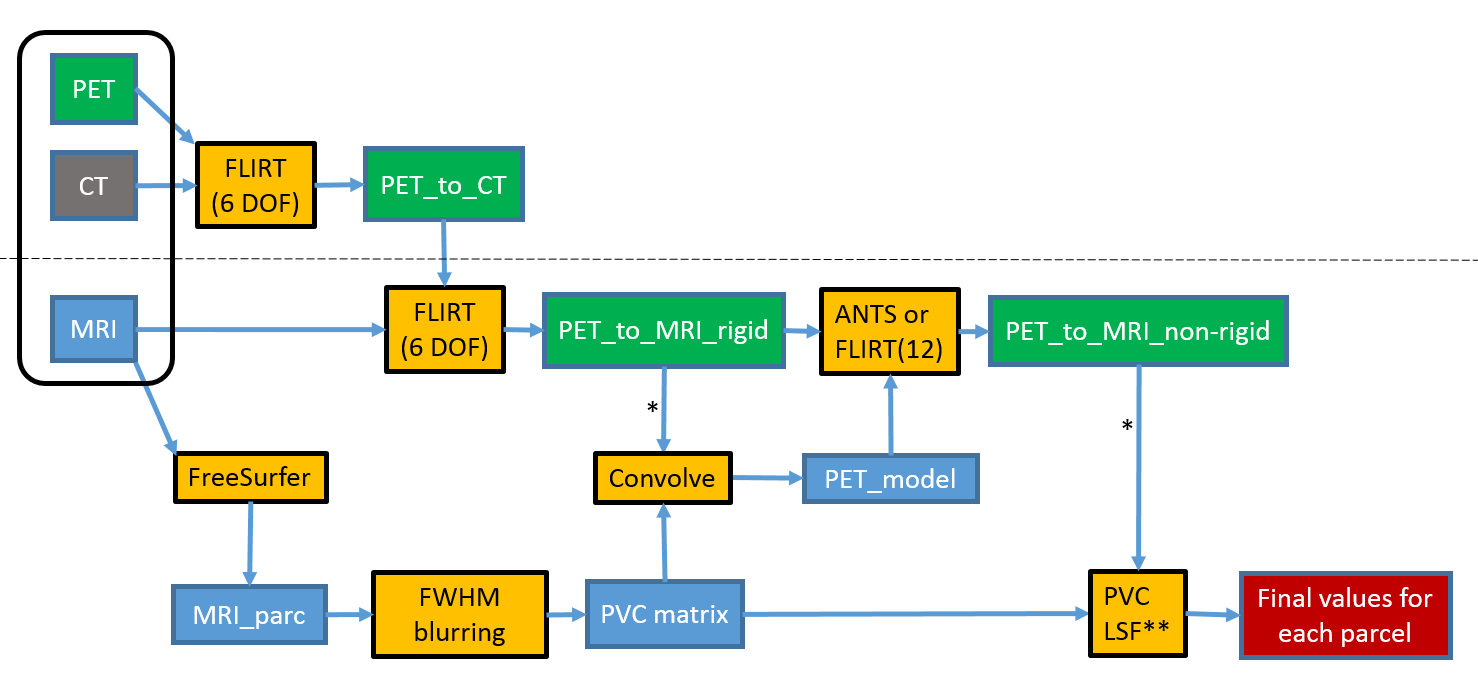


**Supplementary Figure 1.** In the top left box are the three primary image data, which form the inputs into the analysis. PET data are colored in green whereas MRI data are colored in blue. Computational operations are shown by orange boxes. The final numbers used in the paper derive from the red box. All image data below the dashed line are in the native MRI space. The single asterisks denote that only averaged values for each FreeSurfer parcellation are input. The double asterisk denotes the least-square fit is performed either unconstrained or constrained. The box indicating nonrigid coregistration was performed using two methods (nonlinear ANTS or affine FLIRT with 12 degrees of freedom). DOF, degrees of freedom; LSF, least-square fit; PVC, partial volume correction.


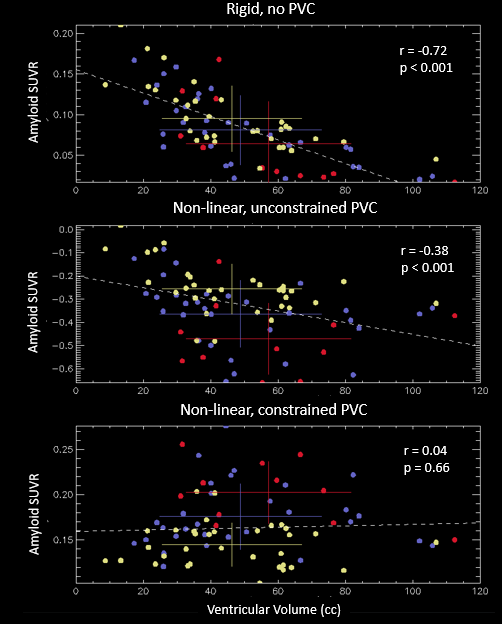


**Supplementary Figure 2.** These three scatter plots are similar to those shown in the main body of the paper, **Figure 2**, except all three are data from the lateral ventricles using variants of model: (top) rigid coregistration with no PVC, (middle) nonlinear coregistration with unconstrained PVC, and (bottom) nonlinear coregistration with constrained PVC. The model used in the bottom figure was that employed in the final analysis of results in this paper. The first two plots show a statistically significant correlation between ventricular SUVR and ventricle volume, which is nonphysical. Only the last model shows a correlation that is zero.

| **Method** | **FWHM 6** | **FWHM 8** | **FWHM Best** |
| --- | --- | --- | --- |
| Entire brain | | | |
| Rigid, no PVC | 0.390 | 0.388 | 0.387 |
| Affine, PVC | 0.250 | 0.220 | 0.220 |
| Nonlinear, PVC | 0.178 | 0.163 | 0.163 |
| Cortex only | | | |
| Rigid, no PVC | 0.304 | 0.325 | 0.304 |
| Affine, PVC | 0.192 | 0.170 | 0.170 |
| Nonlinear, PVC | 0.144 | 0.140 | 0.138 |

**Supplementary Table S1.** PET and MRI coregistration goodness-of-fit results for different methods, averaged across all patients. Abbreviations: FWHM, full width at half maximum; MRI, magnetic resonance imaging; PET, positron emission tomography; PVC, partial volume correction.

| **Method** | **Ratio of Cortical to Subjacent White Matter Amyloid Uptake** | | | |
| --- | --- | --- | --- | --- |
|  | **All Patients** | **MoCA Score ≤ 15** | **MoCA Score > 15** | ***p* Value** |
| Rigid, no PVC | 0.83 | 0.90 | 0.81 | 2.8 × 10^–5^ |
| Affine, PVC | 0.89 | 1.05 | 0.85 | 4.2 × 10^–5^ |
| Nonlinear, PVC | 0.87 | 1.05 | 0.84 | 1.3 × 10^–4^ |

**Supplementary Table S2.** Ratio of cortical to subjacent white matter amyloid uptake for all patients, for patients with lower MoCA scores, and for patients with higher MoCA scores. Abbreviations: MoCA, Montreal Cognitive Assessment; PVC, partial volume correction.
